# Calcineurin is an important factor involved in glucose uptake in human adipocytes

**DOI:** 10.1007/s11010-017-3261-0

**Published:** 2018-01-27

**Authors:** Ana Catarina R. G. Fonseca, Eugénia Carvalho, Jan W. Eriksson, Maria J. Pereira

**Affiliations:** 1 Department of Medical Sciences, University of Uppsala, 751 85 Uppsala, Sweden; 2 Center of Neuroscience and Cell Biology, University of Coimbra, 3004-504 Coimbra, Portugal; 3 The Portuguese Diabetes Association (APDP), 1250-203 Lisbon, Portugal; 4 Department of Geriatrics, University of Arkansas for Medical Sciences, Little Rock, AR 72202 USA; 5 Arkansas Children’s Research Institute, Little Rock, AR 72202 USA

**Keywords:** Diabetes, Calcineurin inhibitors, Adipose tissue, Adipocytes, Glucose uptake, Gene expression

## Abstract

Calcineurin inhibitors are used in immunosuppressive therapy applied after transplantation, but they are associated with major metabolic side effects including the development of new onset diabetes. Previously, we have shown that the calcineurin inhibiting drugs tacrolimus and cyclosporin A reduce adipocyte and myocyte glucose uptakes by reducing the amount of glucose transporter type 4 (GLUT4) at the cell surface, due to an increased internalization rate. However, this happens without alteration in total protein and phosphorylation levels of key proteins involved in insulin signalling or in the total amount of GLUT4. The present study evaluates possible pathways involved in the altered internalization of GLUT4 and consequent reduction of glucose uptake provoked by calcineurin inhibitors in human subcutaneous adipose tissue. Short- and long-term treatments with tacrolimus, cyclosporin A or another CNI deltamethrin (herbicide) decreased basal and insulin-dependent glucose uptake in adipocytes, without any additive effects observed when added together. However, no tacrolimus effects were observed on glucose uptake when gene transcription and protein translation were inhibited. Investigation of genes potentially involved in GLUT4 trafficking showed only a small effect on *ARHGEF11* gene expression (*p* < 0.05). In conlusion, the specific inhibition of calcineurin, but not that of protein phosphatases, decreases glucose uptake in human subcutaneous adipocytes, suggesting that calcineurin is an important regulator of glucose transport. This inhibitory effect is mediated via gene transcription or protein translation; however, expression of genes potentially involved in GLUT4 trafficking and endocytosis appears not to be involved in these effects.

## Introduction

The International Diabetes Federation has shown that the number of people with type 2 diabetes is increasing worldwide. In 2014, there were about 400 million individuals with diabetes mellitus, and this number is expected to increase to approx. 600 million in 2035 [1]. Major clinical trials show that glucose control is not sufficient to prevent comorbidities and its excess mortality, associated with type 2 diabetes [2]. Hence, there is an unmet need to identify and evaluate novel and innovative therapeutic concepts and approaches based on previously unknown molecular pathways.

Calcineurin is a serine/threonine phosphatase controlled by cellular calcium concentrations [3]. Calcineurin has been implicated in a variety of biological responses, including lymphocyte activation and cardiovascular and skeletal muscle development [3]. Since their introduction, calcineurin inhibitors have become the cornerstone of immunosuppressive therapy in solid organ transplantation. However, they are associated with the development of cardiovascular and metabolic complications, like dyslipidemia, hypertension and diabetes melitus [4]. New onset diabetes after transplantation (NODAT) is a common metabolic complication with reported incidence rates up to 50% during the first years after transplantation [5, 6]. Similar to type 2 diabetes, both impaired insulin secretion and insulin resistance in peripheral tissues and liver are the principal pathogenic components of NODAT [7]. However, the mechanisms are not known. Calcineurin inhibitors have been shown to cause adverse effects in white adipose tissue metabolism that can contribute to the development of insulin resistance and diabetes mellitus [8–12]. Our previous work has shown that cyclosporin A and tacrolimus are able to reduce glucose uptakes in human adipocytes and L6 muscle cells. The reduction of glucose uptake was achieved by decreasing the total amount of the glucose transporter type 4 (GLUT4) at the cell surface, mainly due to increased internalization rate [10]. Furthermore, we have also shown that the calcineurin inhibitors increased GLUT4 internalization without affecting total protein levels or phosphorylation of key insulin signalling proteins, including insulin receptor substrate 1 (IRS1), protein kinase B (PKB), AS160 and GLUT4. GLUT4 is the major glucose transporter in muscle and adipose tissue, which constantly cycles between the plasma membrane and intracellular membranes due to the presence of insulin. The GLUT4 endocytic and exocytic itineraries involve a complex interplay of trafficking events and intracellular signalling cascades [13, 14]. Calcineurin can dephosphorylate cytoskeletal proteins, such as actin and tubulin [15–20], and proteins involved in endocytosis, such as dynamin and assembly protein 180 kDa (AP)180 [21]. Also, it is known that calcineurin can induce endocytosis in neurons and other cell types in response to increased cytosolic calcium concentration [21–23].

A role of calcineurin in glucose uptake has also emerged from studies in skeletal muscle in mice. These studies demonstrate that, in transgenic mice overexpressing an activated form of calcineurin, there is an elevation of insulin-stimulated skeletal muscle glucose uptake [24, 25]. Furthermore, several studies have shown that the calcium–calcineurin pathway directly affects insulin-stimulated glucose transport in adipocytes [26, 27] and elevated levels of cytosolic calcium are associated with insulin resistance [28].

Therefore, the aim of the present study was to further investigate possible molecular mechanisms underlying our previous findings, with respect to increased internalization of GLUT4 at the plasma membrane and consequent reduction of glucose uptake induced by calcineurin inhibitors in human subcutaneous adipocytes.

## Subjects and methods

### Subcutaneous adipose tissue (SAT)

Human abdominal SAT biopsies were obtained from nondiabetic subjects (10 males/32 females; age 50 ± 16 years; body mass index (BMI) 26.1 ± 3.2 kg/m^2^). Due to limited amount of adipose tissue obtained, not all experiments were performed in the same biopsies. The number of experiments is indicated in each section below. Subjects were fasted overnight (> 10 h), and fasting venous blood samples were collected in the morning for analysis of glucose, insulin and lipids by routine methods at the Department of Clinical Chemistry at the Sahlgrenska University Hospital and the Uppsala University Hospital. SAT biopsies were performed by needle aspiration of subcutaneous fat from the lower abdomen (*n* = 31) after intradermal local anaesthesia with lidocaine (Xylocain; AstraZeneca, Södertälje, Sweden), or by elective abdominal surgery (*n* = 11) after induction of general anaesthesia.

The clinical and biochemical characteristics of the adipose tissue donors are described in Table 1. Anthropometric measurements including body composition, assessed by bioimpedance, were measured in all subjects [29]. Subjects with diabetes, other endocrine disorders, systemic illnesses or malignancy, as well as ongoing medication with systemic glucocorticoids, beta blockers and immune-modulating therapies were excluded from the study. The study protocol was approved by the Regional Ethics Review Boards in Gothenburg and Uppsala. Written informed consent was obtained from all subjects.

**Table 1:** Clinical and biochemical characteristics of adipose tissue donors (*n* = 42)

Variable	Means	SD
Sex (males/females), *n*	10/32	
Age, years	50	16
Body mass index, kg/m^2^	26.1	3.2
Waist–hip ratio	0.9	0.1
Body fat mass, %	31.0	7.9
Systolic blood pressure, mm Hg	132	18
Diastolic blood pressure, mm Hg	79	12
HbA1c, mmol/mol, IFCC	34.0	3.2
Plasma Glucose, mmol/L	5.4	0.7
Serum Insulin, mU/L	8.0	4.3
HOMA-IR^a^	1.9	1.1
Plasma Triglycerides, mmol/L	1.0	0.4
Plasma Total-Cholesterol, mmol/L	5.3	0.9
Plasma LDL-cholesterol, mmol/L	3.1	0.8
Plasma HDL-cholesterol, mmol/L	1.8	0.6

*HbA1c* glycosylated haemoglobin, *HOMA-IR* homeostatic model assessment-insulin resistance, *LDL* low-density lipoprotein, *HDL* high-density lipoprotein

^a^Calculated as fasting insulin (mU/L) × fasting glucose (mM)/22.5

### Culture of adipose tissue and isolated adipocytes

Adipocytes were isolated from SAT obtained from needle biopsies after collagenase type II digestion (Roche, Mannheim, Germany) in Hank’s medium (Invitrogen Corporation, Paisley, UK) containing 6 mM glucose, 4% BSA and 150 nM adenosine (Sigma Chemical Co., MO, USA) (pH 7.4) for 60 min at 37 °C in a shaking water-bath. Isolated adipocytes were filtered through a 250-μm nylon mesh and pre-incubated for 15 min (short-term) or 20 h (long-term) with tacrolimus (100 nM), cyclosporin A (100 nM), deltamethrin (1 μM), okadaic acid (250 nM), actinomycin D (5 μg/ml) or cycloheximide (25 μM)—alone or in combination (see the Results section).

**Fig. 1:**
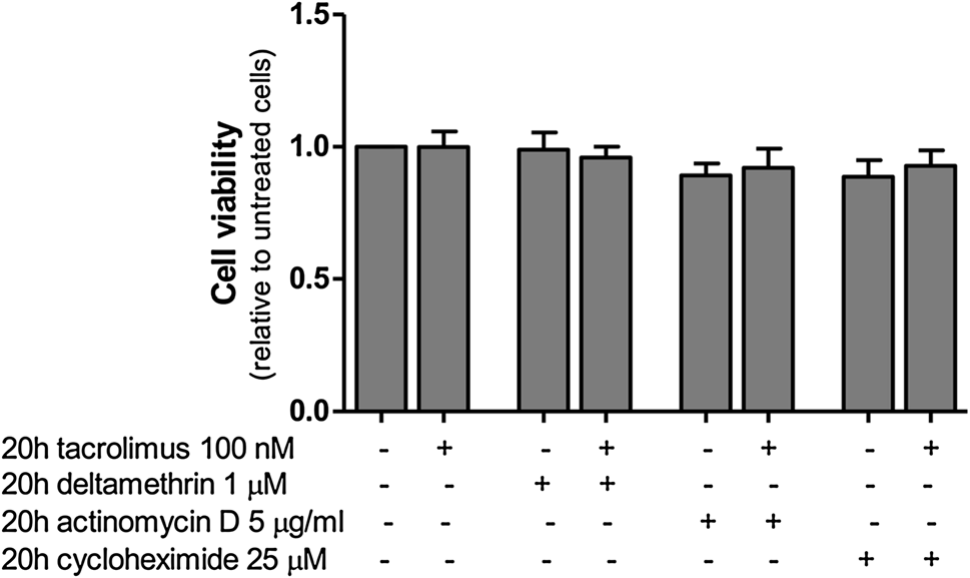
The incubations with tacrolimus, deltamethrin, actinomycin D and cycloheximide do not alter the viability of human subcutaneous adipocytes. After isolation, adipocytes were incubated for 20 h with either tacrolimus 100 nM, deltamethrin 1 μM, actinomycin D 5 μg/ml or cycloheximide 25 μM, and the cell viability was measured. The results were calculated relatively to untreated cell values and represent the means ± SEM of four subjects

The time points and the concentrations were chosen according to previous studies [10, 12, 30–34]. Tacrolimus binds to FK506-binding proteins, and cyclosporin A binds to cyclophilins-forming complexes that inhibit calcineurin [6, 35]. The concentration (100 nM) of tacrolimus and cyclosporin A was previously shown to induce maximum reduction of glucose uptake in adipocytes and to be at therapeutic concentrations commonly used in clinic [10, 12]. Deltamethrin is a type II synthetic pyrethroid insecticide that can also inhibit calcineurin [32], but the mechanism of action is unknown. Deltamethrin was used to test the effect of a different calcineurin inhibitor on glucose uptake for comparison. Actinomycin D and cycloheximide are well-known gene-transcription and protein-translation inhibitors, respectively [33, 34]. They were used to test whether transcription and/or translation is involved in the inhibitory effects of the calcineurin inhibitors on glucose uptake. The concentrations of deltamethrin, actinomycin D and cycloheximide were shown to maximally inhibit calcineurin, gene transcription and protein translation, respectively, without significantly reducing cell viability [32–34] (Fig. 1). Okadaic acid is a phosphatase inhibitor that, at 250 nM concentration, can inhibit the phosphorylated myosin light-chain (PMLC) phosphatase, phosphatase 1 and phosphatase 2A, but not calcineurin (protein phosphatase 2B) [30, 31] .

For short-term incubations, isolated adipocytes were washed three times in glucose-free Krebs Ringer media (KRH) supplemented with 4% BSA, 150 nM adenosine and pH 7.4. Adipocytes were then diluted ten times in supplemented KRH medium and pre-incubated for 15 min with the described conditions for further glucose uptake analysis. For long-term incubations, isolated adipocytes were washed three times in Hank’s medium that contained 6 mM glucose, 4% BSA and 150 nM adenosine and placed in DMEM (Invitrogen) with 6 mM glucose and 10%  FCS (Invitrogen) in the different conditions described and at 37 °C under a gas phase of 5% CO_2_ in a culture chamber for 20 h. After incubation, cells were washed and diluted ten times in KRH medium (4% BSA, 150 nM adenosine, pH 7.4) for further glucose uptake analysis. The average cell diameter was measured in isolated adipocytes from all subjects [36].

Effect of long-term incubation (20 h) with tacrolimus on gene expression of possible intermediates of GLUT4 trafficking was analysed in SAT samples. For this, 100 mg of adipose tissue explants were incubated for 20 h without or with tacrolimus (100 nM) in 24 well polystyrene plates containing 1 ml of DMEM (6 mM glucose, 10% FCS) (Invitrogen Corporation, Paisley, USA) in a humidified atmosphere of 5% CO_2_ at 37 °C. Adipose tissue was thereafter snap-frozen for gene expression analysis.

### Assessment of cell viability

After 20 h incubation of subcutaneous adipocytes (*n* = 4) with tacrolimus (100 nM), cyclosporin A (100 nM), deltamethrin (1 μM), okadaic acid (250 nM), actinomycin D (5 μg/ml) or cycloheximide (25 μM), cell viability was assessed with the water soluble tetrazolium-colorimetric reagent (WST-1, Roche, Mannheim, Germany) according to manufacturer instructions. The viability of adipocytes was not significantly affected with any treatment compared with untreated cells (Fig. 1).

### Glucose uptake in adipocytesz

Glucose uptake in isolated subcutaneous adipocytes was performed according to a previously validated technique for human adipocytes, which reflects rate of transmembrane glucose transport [37]. Briefly, after long-term (20 h) or short-term (15 min) incubation without changing the media, adipocytes were incubated with or without insulin (25 and 1000 mU/ml, Actrapid, NovoNordisk, Bagsvaerd, Denmark) for 15 min, followed by an additional 45 min of incubation with D-[U–^14^C] glucose (0.26 mCi/L, 0.86 mM, Perkin Elmer, Boston, MA, USA). The reaction was stopped by transferring the cells into pre-chilled vials followed by separation from the medium by centrifugation through Dow Corning Xiameter PMX 200/100cC silicone fluid (BDH Prolabo Chemicals, Leuven, Belgium). Radioactivity associated with the cells was then determined using a scintillation counter. Cellular glucose uptake was calculated using the following formula: Cellular clearance of medium glucose = (cell-associated radioactivity × volume)/(radioactivity of medium × cell number × time). Using this experimental setup, glucose uptake is mainly determined by the rate of transmembrane glucose transport. Adipocyte size and number were measured as described previously [38]. Glucose uptake was normalized per cell number for each experimental condition and expressed relative to control. All experiments were performed in triplicates.

### Adipose tissue gene expression

The aim of the adipose tissue gene expression analyses was to verify whether previously reported effects of calcineurin inhibitors on GLUT4 trafficking by increasing the rate of GLUT4 endocytosis [10], could be due to effects on expression of key genes directly involved with these mechanisms.

Total RNA from adipose tissue was isolated with RNeasy Lipid Tissue Mini Kit (Quiagen, Hilden, Germany), and used for cDNA synthesis using High-Capacity cDNA Reverse Transcriptase kit (Applied Biosystems, CA, USA). The protocol was carried out in accordance with manufacturer’s instructions. Total RNA concentration and purity were measured using the Nanodrop 2000 Spectrophotometer (ThermoFisher Scientific, Rockford, USA). Gene expression was analysed using the QuantStudio™ 3 Real-Time PCR Systems (Applied Biosystems, CA, USA).

First, TaqMan® Array—96-well plates (Applied Biosystems, CA, USA) having four housekeeping genes [18S ribosomal RNA, low-density lipoprotein receptor-related protein 10 (LRP-10), glyceraldehyde-3-phosphate dehydrogenase (GAPDH) and glucuronidase beta (GUSB)] plus 92 selected genes (see Table 2 for the list of the genes)— were used (*n* = 3). The selected genes encode for proteins involved in cytoskeleton organization and potentially in GLUT4 trafficking, specially endocytosis, therefore, putative downstream gene targets of calcineurin signalling. The normalization of the gene expression of the 92 analysed genes was performed with the geometrical mean [(*C*_t_ value of LRP-10 × *C*_t_ value of GUSB)^1/2^] of the *C*_t_ values of the two housekeeping genes, GUSB and LRP-10, with lower coefficient of variation (*CV* = standard deviation of Ct values of all samples/mean of all samples). Subsequently, the expression of each gene was normalized to control, and calculated as a relative fold change (${{\text{2}}^{ - \Delta \Delta {C_{\text{t}}}}}$ method).

**Table 2:** Gene expressions in subcutaneous adipose tissue after 20-h treatment with tacrolimus compared with no treatment (control) and analysed with a PCR microarray (*n* = 3)

Abbreviation	Name	Fold change (tacrolimus/control)	*p* value	General function
*ARHGEF11*	*Rho guanine nucleotide exchange factor (GEF) 11*	*1.29*	*0.42*	*Cytoskeleton assembly and function*
*NCK2*	*NCK adaptor protein 2*	*1.24*	*0.33*	*Cytoskeleton assembly and function*
PAK4	p21 protein (Cdc42/Rac)-activated kinase 4	1.22	0.42	Cytoskeleton assembly and function
*LIMK1*	*LIM domain kinase 1*	*1.22*	0.04*	*Cytoskeleton assembly and function*
CALM1	Calmodulin 1 (phosphorylase kinase, delta)	1.21	0.22	Calcium signalling
ARPC3	Actingnalli protein 2/3 complex, subunit 3, 21 kDa	1.20	0.44	Cytoskeleton assembly and function
FKBP5	FK506-binding protein 5	1.19	0.56	Immunophilin, binds to tacrolimus
*ARPC5*	*Actingnalli protein 2/3 complex, subunit 5, 16 kDa*	*1.17*	0.02*	*Cytoskeleton assembly and function*
*RAB4A*	*RAB4A, member RAS oncogene family*	*1.17*	0.002*	*Exocytosis*
CTTN	Cortactin	1.17	0.56	Cytoskeleton assembly and function
DIAPH1	Diaphanous-related formin 1	1.16	0.50	Cytoskeleton assembly and function
ARPC2	Actingnalli protein 2/3 complex, subunit 2, 34 kDa	1.16	0.34	Cytoskeleton assembly and function
PFN2	Profilin 2	1.16	0.34	Cytoskeleton assembly and function
HIP1	Huntingtin interacting protein 1	1.15	0.44	Endocytosis
BAIAP2	BAI1-associated protein 2	1.14	0.39	Cytoskeleton assembly and function
CLASP1	Cytoplasmic linker associated protein 1	1.13	0.51	Cytoskeleton assembly and function
RDX	Radixin	1.13	0.48	Cytoskeleton assembly and function
GSN	Gelsolin	1.13	0.46	Cytoskeleton assembly and function
WAS	Wiskott–Aldrich syndrome	1.12	0.60	Cytoskeleton assembly and function
CYFIP2	Cytoplasmic FMR1 interacting protein 2	1.12	0.36	Cytoskeleton assembly and function
CLIP2	CAP–GLY domain containing linker protein 2	1.12	0.10	Cytoskeleton assembly and function
WASL	Wiskott–Aldrich syndrome-like	1.11	0.67	Cytoskeleton assembly and function
ARPC1B	Actingnalli protein 2/3 complex, subunit 1B, 41 kDa	1.11	0.24	Cytoskeleton assembly and function
ARAP1	ArfGAP with RhoGAP domain, ankyrin repeat and PH domain 1	1.11	0.57	Vesicular trafficking
*LLGL1*	*Lethal giant larvae homolog 1 (Drosophila)*	*1.10*	0.04*	*Vesicular trafficking/exocytosis*
SNAP23	Synaptosomal-associated protein, 23 kDa	1.10	0.52	Exocytosis
PPP3CB	Protein phosphatase 3, catalytic subunit, beta isozyme	1.10	0.47	Calcineurin catalytic subunit
CRK	v-crk avian sarcoma virus CT10 oncogene homolog	1.10	0.52	Cytoskeleton assembly and function
DSTN	Destrin (actin depolymerizing factor)	1.09	0.39	Cytoskeleton assembly and function
ACTB	Actin, beta	1.09	0.37	Cytoskeletal protein
CALM2	Calmodulin 2 (phosphorylase kinase, delta)	1.09	0.74	Calcium signalling
VAMP2	Vesicle-associated membrane protein 2 (synaptobrevin 2)	1.09	0.37	Exocytosis
DNM2	Dynamin 2	1.08	0.45	Endocytosis
MAP3K11	Mitogen-activated protein kinase kinase kinase 11	1.08	0.33	Cytoskeleton assembly and function
IQGAP1	IQ motif containing GTPase activating protein 1	1.08	0.67	Cytoskeleton assembly and function
EZR	Ezrin	1.07	0.05	Cytoskeleton assembly and function
STX8	Syntaxin 8	1.07	0.51	Exocytosis
AP2B1	Adaptor-related protein complex 2, beta 1 subunit	1.07	0.10	Endocytosis
PIKFYVE	Phosphoinositide kinase, FYVE finger containing	1.07	0.54	Vesicular trafficking
NCK1	NCK adaptor protein 1	1.07	0.70	Cytoskeleton assembly and function
*HGS*	*Hepatocyte growth factor-regulated tyrosine kinase substrate*	*1.07*	0.001*	*Vesicular trafficking and degradation*
CDC42BPA	CDC42-binding protein kinase alpha (DMPK-like)	1.06	0.68	Cytoskeleton assembly and function
PPAP2B	Phosphatidic acid phosphatase type 2B	1.06	0.78	Cytoskeleton assembly and function
AP2A1	Adaptor-related protein complex 2, alpha 1 subunit	1.05	0.61	Endocytosis
SSH1	Slingshot protein phosphatase 1	1.05	0.79	Cytoskeleton assembly and function
CASK	Calcium/calmodulin-dependent serine protein kinase (MAGUK family)	1.05	0.71	Calcium signalling
MAPRE2	Microtubule-associated protein, RP/EB family, member 2	1.03	0.82	Cytoskeleton assembly and function
MSN	Moesin	1.03	0.88	Cytoskeleton assembly and function
ACTR3	ARP3 actin-related protein 3 homolog (yeast)	1.03	0.85	Cytoskeleton assembly and function
CDC42EP3	CDC42 effector protein (Rho GTPase-binding) 3	1.03	0.84	Cytoskeleton assembly and function
ROCK1	Rho-associated, coiled-coil containing protein kinase 1	1.03	0.85	Cytoskeleton assembly and function
PPP3CA	Protein phosphatase 3, catalytic subunit, alpha isozyme	1.02	0.91	Calcineurin catalytic subunit
FNBP1L	Formin-binding protein 1-like	1.02	0.90	Endocytosis
WASF1	WAS protein family, member 1	1.01	0.49	Cytoskeleton assembly and function
VAMP8	Vesicle-associated membrane protein 8	1.01	0.95	Exocytosis
CDC42	Cell division cycle 42	1.01	0.92	Cytoskeleton assembly and function
STMN1	Stathmin 1	1.01	0.96	Cytoskeleton assembly and function
CLASP2	Cytoplasmic linker associated protein 2	1.01	0.97	Cytoskeleton assembly and function
STX4	Syntaxin 4	1.01	0.92	Exocytosis
CLTC	Clathrin, heavy chain (Hc)	1.01	0.97	Endocytosis
RAB5A	RAB5A, member RAS oncogene family	1.00	0.99	Endocytosis
VAMP3	Vesicle-associated membrane protein 3	1.00	1.00	Exocytosis
CFL1	Cofilin 1 (non-muscle)	1.00	0.99	Cytoskeleton assembly and function
MAP4	Microtubule-associated protein 4	0.99	0.94	Cytoskeleton assembly and function
MACF1	Microtubule-actin crosslinking factor 1	0.99	0.96	Cytoskeleton assembly and function
PHLDB2	Pleckstrin homology-like domain, family B, member 2	0.98	0.79	Cytoskeleton assembly and function
ARHGAP6	Rho GTPase activating protein 6	0.98	0.76	Cytoskeleton assembly and function
MID1	Midline 1 (Opitz/BBB syndrome)	0.97	0.84	Cytoskeleton assembly and function
VAMP1	Vesicle-associated membrane protein 1 (synaptobrevin 1)	0.97	0.57	Exocytosis
MAPRE1	Microtubule-associated protein, RP/EB family, member 1	0.97	0.69	Cytoskeleton assembly and function
MYLK	Myosin light-chain kinase	0.97	0.82	Cytoskeletal protein
RHOA	Ras homolog family member A	0.97	0.82	Cytoskeleton assembly and function
ARHGDIB	Rho GDP dissociation inhibitor (GDI) beta	0.96	0.79	Cytoskeleton assembly and function
PLD1	Phospholipase D1, phosphatidylcholine-specific	0.96	0.78	Vesicular trafficking
LIMK2	LIM domain kinase 2	0.94	0.47	Cytoskeleton assembly and function
RAC1	Ras-related C3 botulinum toxin substrate 1 (GTP-binding protein Rac1)	0.94	0.63	Cytoskeleton assembly and function
ACTR2	ARP2 actin-related protein 2 homolog (yeast)	0.94	0.44	Cytoskeleton assembly and function
CDC42EP2	CDC42 effector protein (Rho GTPase-binding) 2	0.94	0.52	Cytoskeleton assembly and function
CALD1	Caldesmon 1	0.93	0.41	Cytoskeleton assembly and function
AAK1	AP2 associated kinase 1	0.93	0.44	Endocytosis
CYFIP1	Cytoplasmic FMR1 interacting protein 1	0.93	0.13	Cytoskeleton assembly and function
ARFIP2	ADP-ribosylation factor interacting protein 2	0.92	0.48	Vesicular trafficking
ARPC4	Actingnalli protein 2/3 complex, subunit 4, 20 kDa	0.92	0.40	Cytoskeleton assembly and function
SORBS1	Sorbin and SH3 domain containing 1	0.92	0.46	Cytoskeleton assembly and function
*VASP*	*Vasodilator-stimulated phosphoprotein*	*0.89*	0.02*	*Cytoskeleton assembly and function*
SSH2	Slingshot protein phosphatase 2	0.89	0.30	Cytoskeleton assembly and function
DNM1	Dynamin 1	0.89	0.49	Endocytosis
CLIP1	CAP-GLY domain containing linker protein 1	0.88	0.06	Cytoskeleton assembly and function
SORT1	Sortilin 1	0.88	0.26	Vesicular trafficking
PAK1	p21 protein (Cdc42/Rac)-activated kinase 1	0.88	0.57	Cytoskeleton assembly and function
*MARK2*	*MAP/microtubule affinity-regulating kinase 2*	*0.87*	*0.48*	*Cytoskeleton assembly and function*
*VAPA*	*VAMP (vesicle-associated membrane protein)-associated protein A*	*0.81*	*0.13*	*Exocytosis*

In italic are the genes selected for standard qRT-PCR

Bold values indicate statistical significance: *p < 0.05

Second, a standard qRT-PCR (*n* = 23) was performed to confirm the gene expressions of the two highest- and the two lowest-expressed genes after tacrolimus treatment (fold change) plus the genes that were statistically different between control and tacrolimus treatment using specific TaqMan gene expression assays [assay on demand: Rho guanine nucleotide exchange factor 11 (*ARHGEF11*, Hs01121959_m1, FAM); NCK adaptor protein 2 (*NCK2*, Hs02561903_s1, FAM); LIM domain kinase 1 (*LIMK1*, Hs00242728_m1, FAM); related protein 2/3 complex, subunit 5 (*ARPC5*, Hs00271722_m1, FAM); RAB4A, member RAS oncogene family (*RAB4A*, Hs01106488_m1, FAM); lethal giant larvae homolog 1 (*LLGL1*, Hs01017181_m1, FAM); hepatocyte growth factor-regulated tyrosine kinase substrate (*HGS*, Hs00610371_m1, FAM); vasodilator-stimulated phosphoprotein (*VASP*, Hs01100128_m1, FAM); MAP/microtubule affinity-regulating kinase 2 (*MARK2*, Hs00997759_m1, FAM); and vesicle-associated membrane protein-associated protein A (*VAPA*, Hs00427749_m1, FAM), Applied Biosystems, CA, USA]. The relative quantification of mRNA levels was plotted as the fold change (${{\text{2}}^{ - \Delta \Delta {C_{\text{t}}}}}$ method) compared with control and normalized to the housekeeping gene GUSB (Hs00939627_m1, VIC) (Applied Biosystems, CA, USA) previously shown to have the lowest coefficient of variation between control and tacrolimus treated samples. Samples were run in duplicates.

### Statistical analysis

Data were expressed relative to control (without treatment) as means ± standard error of the mean (SEM) of measurements performed in duplicate or triplicate. Statistical significance analysis was determined using the two-tailed paired *t* test. Comparisons were performed within the same individual to minimize confounding variables. The differences were considered significant for *p* values < 0.05. Statistical analysis was performed using the GraphPad Prism Software (San Diego, CA, USA).

## Results

### Calcineurin inhibition reduces glucose uptake

**Fig. 2:**
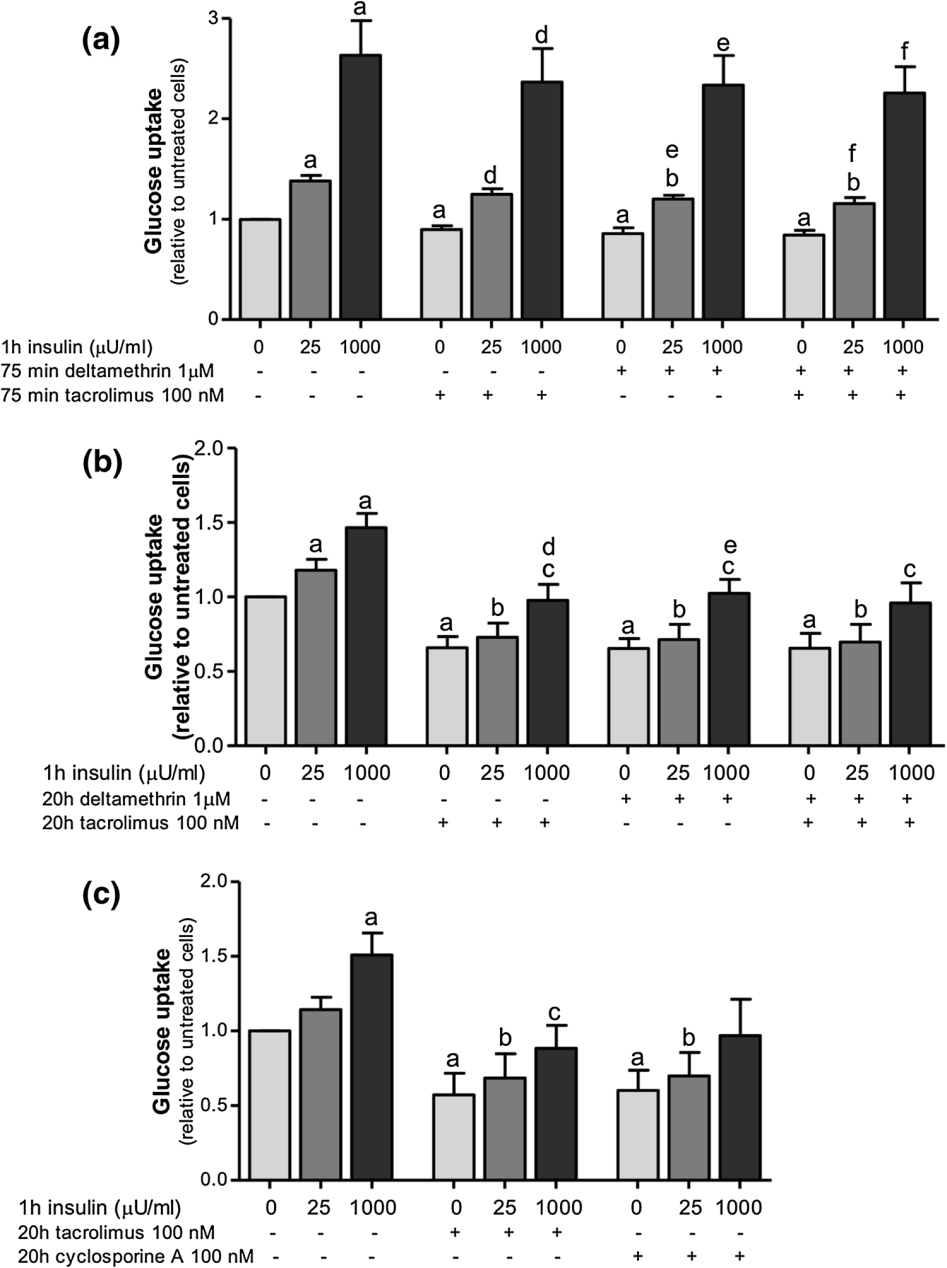
Calcineurin inhibitors decrease glucose uptake in human subcutaneous adipocytes. After isolation, adipocytes were incubated for 75 min (**a**) or 20 h (**b, c**) with 100 nM of tacrolimus (**a**–**c**) and/or 1 μM of deltamethrin (**a, b**) or 100 nM of cyclosporin A (**c**) and the glucose uptake was measured in the absence or presence of 25 or 1000 μU/ml of insulin for 1 h. The results were calculated relatively to untreated cell values and represent the means ± SEM of at least 4 subjects. (*a*) *p* < 0.05 compared with control and no insulin; (*b*) *p* < 0.05 compared with control treated with insulin 25 μU/ml, (*c*) *p* < 0.05 compared with control treated with insulin 1000 μU/ml, (*d*) *p* < 0.05 compared with tacrolimus and no insulin, (*e*) *p* < 0.05 compared with cells treated with deltamethrin and no insulin and (*f*) *p* < 0.05 compared with cells treated with deltamethrin and tacrolimus and no insulin with paired *t* test

Short-term (75 min) or long-term (20 h) incubation of subcutaneous adipocytes with the different calcineurin inhibitors—tacrolimus (100 nM) and deltamethrin (1 μM)—have similar inhibitory effects on basal and insulin-stimulated glucose uptake [short-term by 10–14% (*p* < 0.05) and long-term by 30–60% (*p* < 0.05)], compared with control (Fig. 2a, b). In addition, the long-term incubation of adipocytes with cyclosporin A (100 nM), another calcineurin inhibitor, induced a very similar decrease in glucose uptake compared with tacrolimus (Fig. 2c). The coincubations of adipocytes for 75 min or 20 h with tacrolimus and deltamethrin did not have additive effects on glucose uptake (Fig. 2a, b) suggesting that they may undergo the same mechanism.

The adipogenic differentiated 3T3-L1 cells were also incubated for 20 h with tacrolimus and deltamethrin, but no significant differences were found (data not shown), suggesting that 3T3-L1 cells may not be a good cellular model to represent the effect of calcineurin inhibitors in human adipocytes.

### Inhibition of calcineurin, but not other protein phosphatases, reduces adipocyte glucose uptake

**Fig. 3:**
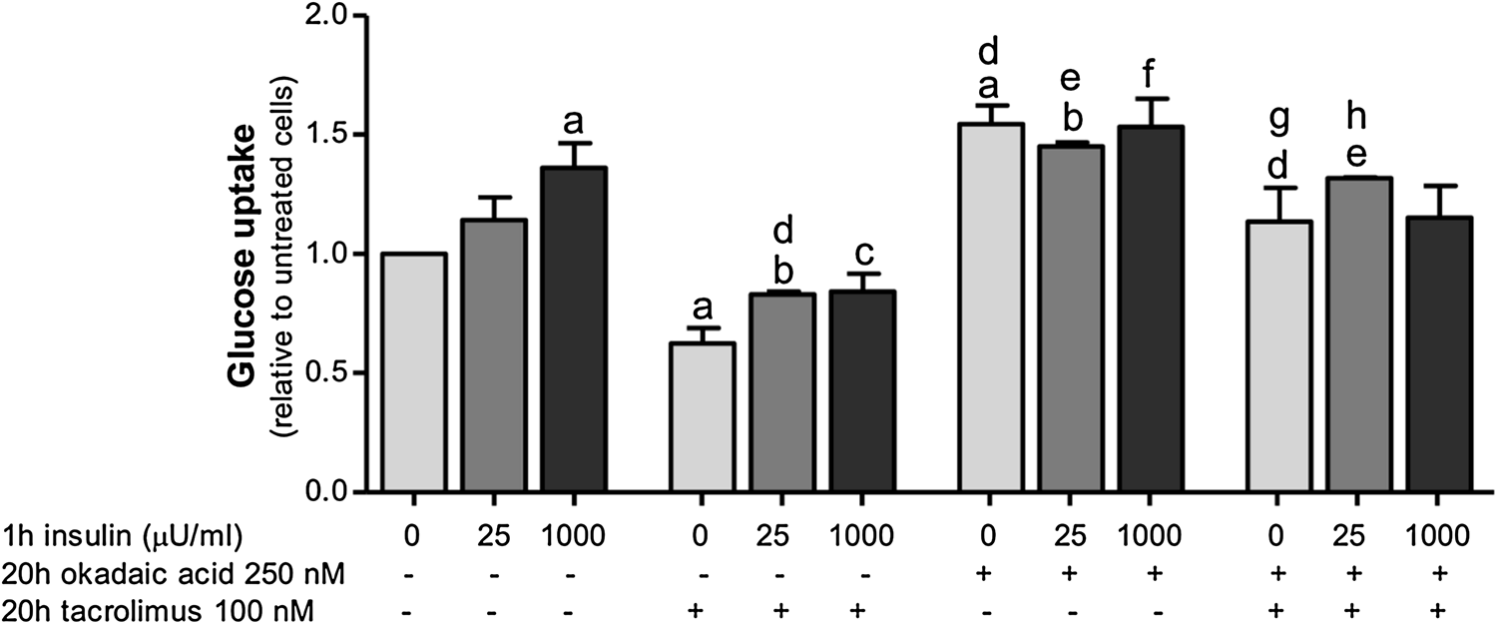
Tacrolimus inhibits okadaic acid-stimulated glucose uptake in human subcutaneous adipocytes. After isolation, adipocytes were incubated for 20 h with 100 nM of tacrolimus and/or 250 nM of okadaic acid and the glucose uptake was measured in the absence or presence of 25 or 1000 μU/ml of insulin for 1 h. The results were calculated relatively to untreated cell values and represent the means ± SEM of at least 4 subjects. (*a*) *p* < 0.05 compared with control and no insulin, (*b*) *p* < 0.05 compared with cells treated with insulin 25 μU/ml, (*c*) *p* < 0.05 compared with cells treated with insulin 1000 μU/ml, (*d*) *p* < 0.05 compared with cells treated with tacrolimus and no insulin, (*e*) *p* < 0.05 compared with cells treated with tacrolimus and insulin 25 μU/ml, (*f*) *p* < 0.05 compared with cells treated with tacrolimus and insulin 1000 μU/ml, (*g*) *p* < 0.05 compared with cells treated with okadaic acid and no insulin, (*h*) *p* < 0.05 compared with cells treated with okadaic acid and insulin 25 μU/ml with paired *t* test

Okadaic acid at 250 nM inhibits protein phosphatases 1 (PP1) and 2A (PP2A), but not calcineurin [30, 31]. Long-term incubation of adipocytes with okadaic acid (250 nM) significantly increased basal and insulin-stimulated glucose uptake by about 50% compared with control (Fig. 3). Addition of tacrolimus decreased basal and insulin-stimulated glucose uptake in the presence of okadaic acid compared with control (Fig. 3) suggesting that okadaic acid and tacrolimus may act through different mechanisms on glucose uptake.

### The inhibitory effect of tacrolimus on glucose uptake requires gene transcription and protein translation

**Fig. 4:**
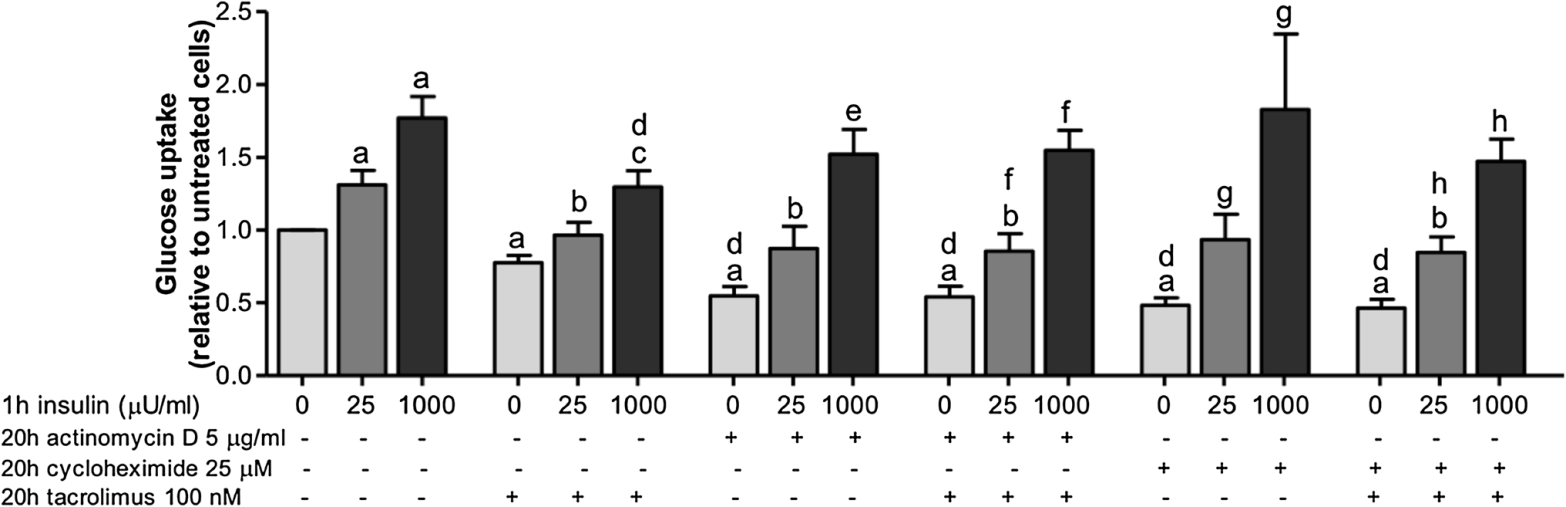
Combinatorial effects of tacrolimus with gene transcription inhibitor or protein-translation inhibitor on adipocyte glucose uptake. After isolation, adipocytes were incubated for 20 h with 5 μg/ml of actinomycin D, 25 μM of cycloheximide and/or 100 nM of tacrolimus and the glucose uptake was measured in the presence or absence of 25 or 1000 μU/ml of insulin for 1 h. The results were calculated relatively to untreated cell values and represent the means ± SEM of at least 6 subjects. (*a*) *p* < 0.05 compared with control and no insulin, (*b*) *p* < 0.05 compared with cells treated with insulin 25 μU/ml, (*c*) *p* < 0.05 compared with cells treated with insulin 1000 μU/ml, (*d*) *p* < 0.05 compared with cells treated with tacrolimus and no insulin, (*e*) *p* < 0.05 compared with cells treated with actinomycin D and no insulin, (*f*) *p* < 0.05 compared with cells treated with actinomycin D and tacrolimus and no insulin, (*g*) *p* < 0.05 compared with cells treated with cycloheximide and no insulin, (*h*) *p* < 0.05 compared with cells treated with cycloheximide and tacrolimus and no insulin with paired *t* test

Long-term (20 h) incubation of adipocytes with the gene transcription inhibitor, actinomycin D (5 μg/ml), and the protein-translation inhibitor, cycloheximide (25 μM), decreased both basal and 25 μU/ml insulin-stimulated glucose uptake by about 30–50% (*p* < 0.05), compared with control (Fig. 4). Addition of tacrolimus did not further affect the basal and insulin-stimulated glucose uptake. This suggests that the inhibitory effect of tacrolimus on glucose uptake might be mediated by the regulation of gene and/or protein expression.

### Effects of tacrolimus on the expression of genes involved in GLUT4 translocation

To evaluate whether tacrolimus could affect expression of genes involved in GLUT4 vesicle translocation, 92 genes were analysed in SAT treated or untreated with tacrolimus for 20 h (*n* = 3, Table 2). These genes correspond to proteins that regulate the cellular cytoskeleton and vesicular trafficking, and might potentially be involved in GLUT4 trafficking, especially endocytosis. *ARHGEF11* (fold change = 1.29, *p* = NS) and *NCK2* (fold change = 1.24, *p* = NS) were the two genes with the greatest increase in gene expression after tacrolimus treatment, compared with non-treated tissue, whereas *VAPA* (fold change = 0.81, *p* = NS) and *MARK2* (fold change = 0.87, *p* = NS) were the two genes with the greatest decrease in gene expression after tacrolimus incubation, compared with control. Furthermore, *LIMK1* (fold change = 1.22, *p* < 0.05), *ARPC5* (fold change = 1.17, *p* < 0.05), *RAB4A* (fold change = 1.17, *p* < 0.01), *LLGL1* (fold change = 1.10, *p* < 0.05) and *HGS* (fold change = 1.07, *p* < 0.01) were significantly increased by tacrolimus treatment, while *VASP* (fold change = 0.89, *p* < 0.05) was significantly decreased by tacrolimus compared with untreated SAT (Table 2).

The expression of these genes was confirmed by qRT-PCR using SAT from a larger cohort (*n* = 23) that was treated in similar conditions (with or without 100 nM tacrolimus for 20 h). Among the genes selected, only *ARHGEF11* was shown to be significantly increased by tacrolimus treatment (Table 3), but with a small effect (fold change = 1.06, *p* < 0.05).

**Table 3:** Gene expression in subcutaneous adipose tissue after treatment with tacrolimus compared with no treatment (control) and analysed by qRT-PCR (*n* = 23)

Gene	Fold change (tacrolimus/control)	SD	*p* value
ARCP5	1.08	0.04	0.08
ARHGEFII	1.06	0.02	0.02*
HGS	1.04	0.04	0.32
LIMKI	0.98	0.05	0.69
LLGLI	1.09	0.06	0.13
MARK2	0.98	0.04	0.59
NCK2	1.12	0.08	0.13
RAB4A	1.02	0.02	0.44
VAPA	1.05	0.03	0.07
VASP	0.98	0.07	0.81

*ARPC5* actingnalli protein 2/3 complex, subunit 5, *ARHGEF11* Rho guanine nucleotide exchange factor (GEF) 11, *HGS* hepatocyte growth factor-regulated tyrosine kinase substrate, *LIMK1* LIM domain kinase 1, *LLGL1* lethal giant larvae homolog 1, *MARK2* MAP/microtubule affinity-regulating kinase 2, *NCK2* NCK adaptor protein 2, *RAB4A* RAB4A, member RAS oncogene family, *VAPA* VAMP (vesicle-associated membrane protein)-associated protein A, *VASP* vasodilator-stimulated phosphoprotein

*p < 0.05

## Discussion

The specific inhibition of calcineurin by tacrolimus, cyclosporin A and deltamethrin, but not the inhibition of other protein phosphatases 1, 2A and phosphorylated myosin light-chain, reduced glucose uptake in subcutaneous adipocytes, suggesting that calcineurin plays an important role in glucose uptake in human, as well as in rodent adipocytes, as previously described [8, 39]. This effect required at least in part gene transcription and/or protein synthesis, as we described. Analysis on the effect of tacrolimus on expression of genes involved in cytoskeleton function and potentially in GLUT4 trafficking suggests that *ARHGEF11* could be a putative downstream gene target of calcineurin signalling associated with GLUT4 trafficking.

Tacrolimus and cyclosporin A have different biochemical structures, but they inhibit calcineurin through similar mechanisms of action: both bind to immunophilins forming a complex in the cytosol that binds and blocks calcineurin [6, 35]. Tacrolimus binds mainly to FK506-binding proteins (FKBP) and cyclosporin A binds to cyclophylins [6]. Both immunophilins interacts with calcineurin in absence of ligands. Deltamethrin is a type II synthetic pyrethroid insecticide known to be a potent specific inhibitor of calcineurin [32]. This is the first study showing important effects of deltamethrin on human adipocyte glucose uptake. In this study, short- or long-term incubation with tacrolimus, cyclosporin A and the alternative calcineurin inhibitor deltamethrin decreased basal and insulin-stimulated glucose uptake in subcutaneous adipocytes in a similar way, indicating that calcineurin plays an important role for regulation of glucose uptake in human adipocytes. Further evidence comes from the lack of additive effect on glucose uptake when coincubating adipocytes with tacrolimus and deltamethrin, suggesting that their effects on glucose uptake may be mediated by the same mechanism, the inhibition of calcineurin.

Okadaic acid inhibits PP1 and PP2A at nanomolar concentrations, but has no effect on calcineurin (PP2B) with the concentration used in this work [30, 31]. Okadaic acid is also known to stimulate adipocyte glucose uptake mainly through PP2A inhibition [6, 40] and independently of phosphoinositide 3-kinase activation [41, 42]. Tacrolimus reduced okadaic acid-stimulated glucose uptake to a similar extent as in control, suggesting that okadaic acid and tacrolimus effects on glucose uptake could be mediated through different pathways.

The degree of inhibition of basal and insulin-stimulated glucose uptake was similar by both calcineurin inhibitors, suggesting that this effect is independent of the early steps of the insulin signalling. These data is in agreement with our previous findings showing that calcineurin inhibitors, tacrolimus and cyclosporin A, decrease glucose uptake in isolated adipocytes by removing GLUT4 from the plasma membrane, via an increased rate of endocytosis [10], but with no apparent defects on insulin signalling including expression and phosphorylation and total protein levels of GLUT4 and GLUT1 [10]. Inhibitory effects of cyclosporin A on adipocyte glucose uptake have also been shown in adipocytes isolated from long-term cyclosporin A-treated rats (up to 9 weeks) or ex vivo treated with cyclosporin A [8, 39]. However, long-term treatment of rats reduces the expression of genes and proteins involved in glucose uptake, such as IRS1 and GLUT4 [39]. Furthermore, overexpression of an activated form of calcineurin in skeletal muscle of mice, induce changes in the expression of genes involved in lipid and glucose metabolism, including GLUT4, with concomitant elevation of insulin-stimulated skeletal muscle glucose uptake [24].

On the other hand, our data suggest that treatment of the 3T3-L1 adipocyte mouse cell line with tacrolimus or deltamethrin does not affect glucose uptake, which is also in agreement with previous findings [43]. Thus, it seems that the effect of the calcineurin inhibitors on glucose uptake might vary between species and exposure times and therefore it is important to use a human model to study the mechanisms involved in calcineurin inhibition on glucose uptake.

Coincubation of adipocytes with the gene transcription inhibitor, actinomycin D, or with the protein-translation inhibitor, cycloheximide, and tacrolimus prevented the inhibitory effect of tacrolimus on glucose uptake. This suggests that gene transcription and/or protein translation are required and important for the inhibitory effect of tacrolimus on glucose uptake. Furthermore, the inhibitory effects of the calcineurin inhibitors were more evident with longer (20 h) than with shorter pre-incubation time (15 min), suggesting that the calcineurin inhibitors are more likely to affect gene and/or protein expression rather than acute phosphorylation events. This supports the hypothesis that calcineurin is an important factor involved in glucose uptake in human adipocytes and this effect likely requires gene transcription and protein synthesis.

In the current analysis, we evaluated effects of calcineurin inhibition on the expression of genes that encode proteins involved in the regulation of cytoskeleton and potentially in GLUT4 trafficking (endocytosis and exocytosis) by gene microarray in SAT explants previously treated with tacrolimus (*n* = 3). The genes with the greatest increase (*ARHGEF11* and *NCK2*) and greatest decrease (*VAPA* and *MARK2*) after tacrolimus treatment, and genes significantly affected by tacrolimus treatment (*LIMK1, ARPC5, RAB4A, LLGL1, HGS* and *VASP*) were further analysed using a larger cohort of subjects (*n* = 23). However, only *ARHGEF11* was significantly increased by chronic tacrolimus treatment. Some variants of *ARHGEF11* have been associated with type 2 diabetes and schizophrenia in several ethnic populations [44–47]. *ARHGEF11* acts as a guanine nucleotide exchange factor for RhoA GTPase and mediates the interaction with the actin cytoskeleton [48]. It is involved in the regulation of G protein signalling, actin cytoskeletal organization [49] and other processes such as insulin signalling [50], insulin secretion [51], and lipid metabolism [52]. Nevertheless, the increase in *ARHGEF11* in gene expression of about 6% found in this study is unlikely to have biological relevance and explain the differences shown on glucose uptake. Altogether our data suggest that the inhibitory effects of calcineurin inhibitors on glucose uptake likely requires gene transcription and protein synthesis, but not the expression of the studied genes potentially involved in GLUT4 trafficking and glucose uptake. However, it could include effects on other genes yet unstudied. Hence, more work is needed to find the mechanisms involved in glucose uptake inhibition by calcineurin inhibitors and more importantly identifying the mechanism of calcineurin regulation on glucose uptake in adipose tissue.

In conclusion, the specific inhibition of calcineurin by tacrolimus, cyclosporin A or deltamethrin, decreased glucose uptake in human subcutaneous adipocytes, suggesting that calcineurin is an important mechanism in the regulation of glucose transport. This effect likely requires gene transcription and protein synthesis, but not via effects on GLUT4 or classical genes known to regulate vesicular trafficking, such as dynamin and RAB proteins. These data suggest that calcineurin is an important regulator of glucose uptake in human adipocytes and its inhibition might contribute to impaired glucose handling in peripheral tissues, as reported with calcineurin modifying therapy in organ-transplanted patients.
